# Serum C-reactive protein and albumin are useful biomarkers for tight control management of Crohn’s disease in Japan

**DOI:** 10.1038/s41598-020-57508-7

**Published:** 2020-01-16

**Authors:** Hisashi Shiga, Izuru Abe, Motoyuki Onodera, Rintaro Moroi, Masatake Kuroha, Yoshitake Kanazawa, Yoichi Kakuta, Katsuya Endo, Yoshitaka Kinouchi, Atsushi Masamune

**Affiliations:** 1 Division of Gastroenterology, Tohoku University Graduate School of Medicine, Sendai, Japan; 2 Division of Gastroenterology and Hepatology, Tohoku Medical and Pharmaceutical University, Sendai, Japan; 3 Health Administration Center, Center for the Advancement of Higher Education, Tohoku University, Sendai, Japan

**Keywords:** Prognostic markers, Crohn's disease

## Abstract

Tight control management of Crohn’s disease (CD) based on biomarkers is more effective than conventional clinical management; however, fecal calprotectin is not allowed in Asian and some Western countries. To investigate whether tight control management based on readily available serum biomarkers results in better outcomes, we retrospectively reviewed treatment courses of consecutive Japanese CD patients treated with anti-tumor necrosis factor agents between 2003 and 2018. The association between failure of tight control (C-reactive protein (CRP) ≥ 0.5 mg/dL or albumin (Alb) < 3.8 g/dL at week 8 or 24) and subsequent major adverse outcomes (MAOs; hospitalization related to CD worsening, surgery, and discontinuation due to treatment failure) were analyzed. Among 223 patients followed for >8 weeks, 88 patients experienced MAOs. Multivariate analysis identified penetrating type, CRP ≥ 0.5 mg/dL and Alb < 3.8 g/dL at week 8 as independent risk factors (hazard ratios: 2.16, 2.06, and 2.08, respectively). Among 204 patients followed for >24 weeks, 80 patients experienced MAOs. Penetrating type, CRP ≥ 0.5 mg/dL, and Alb < 3.8 g/dL at week 24 were identified as independent risk factors (2.39, 1.90, and 2.20, respectively). Even in settings without fecal calprotectin, tight control management based on serum CRP and Alb may help avoid MAOs.

## Introduction

Crohn’s disease (CD) is a chronic inflammatory bowel disease characterized by recurrent episodes of relapse and remission. The conventional treatment approach guided by clinical symptoms may lead to unfavorable prognosis and adverse outcomes such as stenosis or fistula. These intestinal complications necessitate surgery in a large proportion of patients; cumulative surgery rates of 16.3% at 1 year, 33.3% at 5 years, and 46.6% at 10 years following diagnosis have been reported^1^.

Recent studies have identified mucosal healing as an important marker of disease activity and prognosis^2,3^. Mucosal healing was shown to be associated with lower cumulative surgery rate and better prognosis^4^. Disease monitoring and treatment optimization aimed at mucosal healing may improve disease prognosis. Based on these reports, a treat-to-target approach for CD has been advocated as a strategy to improve prognosis and minimize the need for surgery; this approach entails the use of both clinical and endoscopic remission as indicators^5^. However, in contrast to patients with ulcerative colitis in whom lesions are limited to colorectum and sigmoidoscopy is enough for assessing disease activity, it is not easy to perform frequent ileocolonoscopy or enteroscopy in patients with CD who have intermittent and sometimes extensive lesions throughout the intestines. Therefore, biomarker remission, as indicated by normalization of C-reactive protein (CRP) and fecal calprotectin, was considered as an adjunctive or alternative target^5^.

The efficacy of the treat-to-target approach using biomarkers was assessed in a randomized controlled trial (the so-called CALM study)^6^. In the clinical management group, the treatment strategy was based on clinical symptoms only, while patients in the tight control group were managed based on clinical symptoms together with serum or fecal biomarkers; treatment failure criteria was set at CRP > 5 mg/L or fecal calprotectin >250 µg/g. After 48 weeks, tight control management was found to be more effective than conventional clinical management. In that study, fecal calprotectin was the most important index and in >90% of cases, treatment intensification was based on fecal calprotectin exceeding the treatment failure criteria. However, there is a need to develop additional biomarkers because fecal calprotectin is not always readily available in routine clinical practice in Asian and some Western countries. Serum CRP has been shown to be associated with disease activity in CD^7,8^; however, it has not been proven to be a reliable serum biomarker of tight control in Asian settings with different susceptibility genes and clinical characteristics from Western countries^9^. Moreover, another critical issue is inadequate data on optimal standards of biomarkers including serum CRP.

In this study, we sought to investigate whether tight control management based only on serum biomarkers that can be used in routine clinical practice leads to better outcome, and to identify their optimal standards for tight control management in Japanese patients with CD who were treated with anti-tumor necrosis factor (TNF) agents.

## Materials and Methods

### Subjects

We reviewed the treatment course of consecutive patients with CD who were naïve to biologics and were treated with anti-TNF agents at the Tohoku University Hospital between July 2003 and September 2018. CD was diagnosed based on the diagnostic criteria proposed by the Research Group of Intractable Inflammatory Bowel Disease under the aegis of the Ministry of Health, Labor and Welfare of Japan^10^. Patients were included irrespective of the disease location or disease behavior. We excluded patients who discontinued treatment with anti-TNF agents for some reasons within 8 weeks. The Ethics Committee of the Tohoku University Hospital approved the study; the clinical procedures were carried out in accordance with the Declaration of Helsinki. Written informed consent was obtained from all patients.

### Treatment courses

Patients were treated with anti-TNF agents (infliximab or adalimumab). We administered 5 mg/kg of infliximab at weeks 0, 2, and 6, and then continued the same dose (5 mg/kg) every 8 weeks. Patients who exhibited loss of response were managed by dose optimization of infliximab (dose increase to 10 mg/kg or interval shortening to every 4 weeks)^11^. Adalimumab was administered as 160 mg at week 0, 80 mg at week 2, 40 mg at week 4, and then continued at the same dose (40 mg) every 2 weeks. Patients who exhibited loss of response were managed by dose optimization of adalimumab (dose increase to 80 mg)^11^. In Japan, neither interval shortening of adalimumab to every week nor measurement of serum anti-TNF trough level are covered under the health insurance system.

We reviewed the results of blood investigations, and defined the indices of tight control based on the upper quartile of serum CRP and the lower quartile of serum albumin (Alb) at week 24. The achievement of tight control was judged at week 8 or 24 in each case.

### Primary and secondary outcomes

The primary outcome was major adverse outcomes (MAOs: hospitalization related to CD worsening, surgery, and discontinuation due to treatment failure). The association between failure to achieve tight control at week 8 or 24 and subsequent MAOs were analyzed. The secondary outcomes were clinical factors (gender, age at diagnosis, disease duration, disease location, disease behavior, anal lesions, smoking habit or concomitant immunosuppressants) and serum biomarkers (CRP and Alb at week 8 or 24) affecting the primary outcome.

### Statistical analysis

Data are presented as median value and interquartile range (IQR). Between-group differences were assessed using the Chi-squared test or Fisher’s exact probability test, as appropriate. The cumulative rate of MAOs was calculated using the Kaplan-Meier method, and between-group differences were assessed using the log-rank test. To identify factors affecting primary outcome, we also performed multivariate analyses using Cox proportional hazards model with clinical characteristics and serum biomarkers as covariates. The JMP Ver. 11 software (SAS Institute Inc., Cary, NC, USA) was used to perform all statistical analyses. *P* values less than 0.05 were considered indicative of statistically significant difference.

## Results

### Clinical characteristics and medical treatments

A total of 245 patients were treated with anti-TNF agents (185 with infliximab and 60 with adalimumab). Of these, 22 patients were excluded because of relocation or surgery due to treatment failure within 8 weeks from the start of anti-TNF therapy. Thus, the treatment course was continued for more than 8 weeks by 223 patients; of these, 204 patients continued the treatment course for more than 24 weeks.

Table 1 shows the clinical characteristics of 223 patients. These included 159 males (71.3%) and 64 females (28.7%); the median age at diagnosis was 21 years (IQR 25–75%: 18–27) and the median disease duration at the start of anti-TNF therapy was 6 years (IQR 25–75%: 1–12). Seventy-five patients (33.6%) were under the age of 17 years at diagnosis; 148 patients (66.4%) had disease duration of 3 years or more. The disease type included 32 ileitis type (14.3%), 159 ileocolitis type (71.3%), and 32 colitis type (14.3%). The disease behavior included 79 inflammatory type (35.4%), 93 stricturing type (41.7%), and 51 penetrating type (22.9%); 139 patients (62.3%) had anal lesions. Ninety-seven patients (43.5%) had smoking habit (current smokers, 47; past smokers, 50). At the start of anti-TNF therapy, 5-aminosalicilic acid, prednisolone, immunosuppressants (azathioprine or 6-mercaptopurine) and elemental nutrition were concomitantly used in 186 (83.4%), 43 (19.3%), 40 (17.9%), and 70 (31.4%) patients, respectively.

**Table 1:** Clinical characteristics at the start of anti-TNF agents.

Clinical characteristics		N = 223
Gender		
Male		159 (71.3%)
Female		64 (28.7%)
Age at diagnosis	Median years (IQR 25–75%)	21 (18–27)
<17 years		46 (20.6%)
≥17 years		177 (79.4%)
Age at the start of anti-TNF agents	Median years (IQR 25–75%)	31 (23–38)
Disease duration	Median years (IQR 25–75%)	6 (1–12)
<3 years		75 (33.6%)
≥3 years		148 (66.4%)
History of intestinal resection		
Once or more		92 (41.3%)
Never		131 (58.7%)
Disease type		
Ileitis type		32 (14.3%)
Ileocolitis type		159 (71.3%)
Colitis type		32 (14.3%)
Disease behavior		
Penetrating type		51 (22.9%)
Stricturing type		93 (41.7%)
Inflammatory type		79 (35.4%)
Anal lesions		
Present		139 (62.3%)
Absent		84 (37.7%)
Extraintestinal manifestations		
Present		45 (20.2%)
Absent		178 (79.8%)
History of smoking		
Current smoker		47 (21.1%)
Past smoker		50 (22.4%)
Never		111 (49.8%)
Unknown		15 (6.7%)
Anti-TNF agents		
Infliximab		168 (75.3%)
Adalimumab		55 (24.7%)
Concomitant therapy^†^		
5-ASA		186 (83.4%)
Prednisolone		43 (19.3%)
Immunosuppressants		40 (17.9%)
Elemental diet		70 (31.4%)
Elemental diet (>900 kcal/day)		27 (12.1%)

5-ASA: 5-aminosalicylic acid; IQR: interquartile range; TNF: tumor necrosis factor.

^†^includes overlapping cases.

### Treatment course with anti-TNF agents

At the start of anti-TNF therapy, median serum CRP and Alb levels were 0.6 mg/dL (IQR 25–75%: 0.1–2.1) and 3.7 g/dL (IQR 25–75%: 3.2–4.0), respectively. Infliximab was administered to 168 patients (75.3%), while 55 patients were administered adalimumab (24.7%). Owing to loss of response during the treatment course, 41 patients were managed by optimization of treatment: increase in dose of infliximab to 10 mg/kg in 20 patients; increase in dose of adalimumab to 80 mg in 5 patients; addition of prednisolone in 2 patients; addition of immunosuppressants in 14 patients (azathioprine in 12 patients and 6-mercaptopurine in 2 patients).

During treatment with anti-TNF therapy, median serum CRP level improved to 0.1 mg/dL (IQR 25–75%: 0.1–0.2) and 0.1 mg/dL (IQR 25–75%: 0.1–0.4) at weeks 8 and 24, respectively. In the present study, index of tight control of CRP level corresponded to 0.4 mg/dL or less (<0.5 mg/dL) based on the upper quartile. Regarding CRP level at week 8, 188 patients (84.3%) achieved tight control, while 35 patients (15.7%) had CRP levels ≥ 0.5 mg/dL. Regarding CRP at week 24, 158 patients (77.5%) achieved tight control, while 46 patients (22.5%) had CRP levels ≥ 0.5 mg/dL.

Similarly, median Alb level improved to 4.1 g/dL (IQR 25–75%: 3.8–4.4) and 4.1 g/dL (IQR 25–75%: 3.8–4.4) at weeks 8 and 24, respectively. Therefore, index of tight control in Alb level corresponded to ≥3.8 g/dL based on the lower quartile. Regarding Alb level at week 8, 169 patients (75.8%) achieved tight control, while 54 patients (24.2%) had Alb levels < 3.8 g/dL. Regarding Alb level at week 24, 159 patients (77.9%) achieved tight control, while 45 patients (22.1%) had Alb levels < 3.8 g/dL.

### Primary and secondary outcomes

Among the 223 patients who were followed up for >8 weeks [median follow-up: 1,424 days (IQR 25–75%: 504–2527)], 88 patients experienced MAOs (hospitalization, 77 patients; surgery, 37 patients; discontinuation due to treatment failure, 41 patients). Among the 204 patients who were followed up for >24 weeks [median follow-up: 1,688.5 days (IQR 25–75%: 683–2582)], 80 patients experienced MAOs (hospitalization, 69 patients; surgery, 31 patients; discontinuation due to treatment failure, 38 patients).

**Figure 1:**
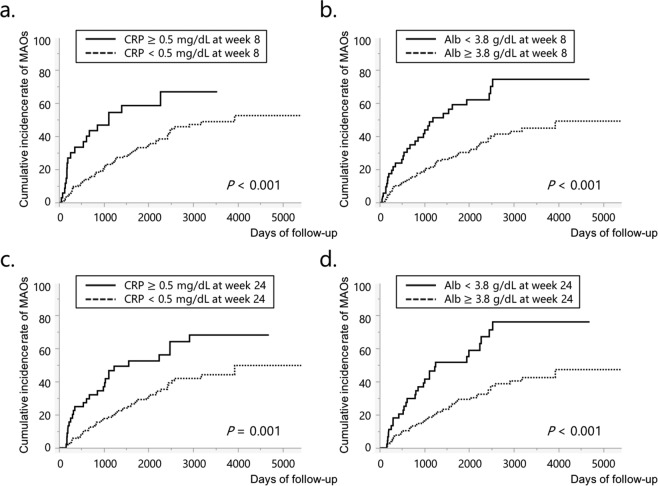
The cumulative incidence rates of major adverse outcomes. When comparing groups divided according to serum CRP or Alb levels at week 8 or 24, the cumulative incidence rate was significantly higher in the patients with CRP ≥ 0.5 mg/dL at week 8 (**a**), Alb < 3.8 g/dL at week 8 (**b**), CRP ≥ 0.5 mg/dL at week 24 (**c**), and Alb < 3.8 g/dL at week 24 (**d**). Alb: albumin; CRP: C-reactive protein; MAOs: major adverse outcomes (hospitalization related to worsening of Crohn’s disease, surgery, and discontinuation of anti-tumor necrosis factor agents due to treatment failure).

On univariate analyses, the rates of MAOs were significantly higher in patients with penetrating type disease, CRP ≥ 0.5 mg/dL, and Alb < 3.8 g/dL at week 8 (Table 2, Fig. 1a,b). We performed multivariate analysis of the cumulative rate of MAOs with clinical characteristics as covariates; the results showed that penetrating type disease, CRP ≥ 0.5 mg/dL, and Alb < 3.8 g/dL at week 8 were independent risk factors for MAOs (hazard ratios: 2.16, 2.06, and 2.08, respectively). No other factors were identified as significant risk factors (Table 2).

**Table 2:** Possible risk factors for major adverse outcomes among clinical characteristics and serum biomarkers at week 8.

Clinical characteristics	N = 223	Univariate		Multivariate		
		MAOs	*P*	HR	95% CI	*P*
Gender						
Male	159 (71.3%)	63 (39.6%)	0.57	1.08	0.65–1.85	0.76
Female	64 (28.7%)	25 (39.1%)		1.00		
Age at diagnosis						
<17 years	46 (20.6%)	19 (41.3%)	0.83	0.99	0.56–1.71	0.99
≥17 years	177 (79.4%)	69 (39.0%)		1.00		
Disease duration						
<3 years	75 (33.6%)	27 (36.0%)	0.72	1.30	0.74–2.24	0.36
≥3 years	148 (66.4%)	61 (41.2%)		1.00		
Disease type						
Ileitis type	32 (14.3%)	11 (34.4%)	0.64	0.89	0.37–2.16	0.80
Ileocolitis type	159 (71.3%)	66 (41.5%)		1.06	0.56–2.17	0.87
Colitis type	32 (14.3%)	11 (34.4%)		1.00		
Disease behavior						
Penetrating type	51 (22.9%)	27 (52.9%)	0.02	2.16	1.14–4.14	0.02
Stricturing type	93 (41.7%)	37 (39.8%)		1.33	0.73–2.48	0.36
Inflammatory type	79 (35.4%)	24 (30.4%)		1.00		
Anal lesions						
Present	139 (62.3%)	56 (40.3%)	0.81	0.98	0.62–1.59	0.94
Absent	84 (37.7%)	32 (38.1%)		1.00		
History of smoking						
Present	47 (21.1%)	20 (42.6%)	0.78	1.03	0.58–1.78	0.91
Others	176 (78.9%)	68 (38.6%)		1.00		
Immunosuppressants						
Present	40 (17.9%)	17 (42.5%)	0.73	1.05	0.59–1.77	0.85
Absent	183 (82.1%)	71 (38.8%)		1.00		
CRP at week 8						
≥0.5 mg/dL	35 (15.7%)	19 (45.7%)	<0.01	2.06	1.15–3.55	0.02
<0.5 mg/dL	188 (84.3%)	69 (36.7%)		1.00		
Alb at week 8						
<3.8 g/dL	54 (24.2%)	31 (57.4%)	<0.01	2.08	1.27–3.37	<0.01
≥3.8 g/dL	169 (75.8%)	57 (33.7%)		1.00		

Alb: albumin; CI: confidence interval; CRP: C-reactive protein; HR: hazard ratio; MAOs: major adverse outcomes (hospitalization related to worsening of Crohn’s disease, surgery, and discontinuation due to treatment failure); TNF: tumor necrosis factor.

Similar to the above results, the rates of MAOs were significantly higher in patients with penetrating type disease, CRP ≥ 0.5 mg/dL, and Alb < 3.8 g/dL at week 24 (Table 3, Fig. 1c,d). On multivariate analysis, penetrating type disease, CRP ≥ 0.5 mg/dL, and Alb < 3.8 g/dL at week 24 were independent risk factors for MAOs (hazard ratios: 2.39, 1.90, and 2.20, respectively). There were no significant differences regarding other factors (Table 3).

**Table 3:** Possible risk factors for major adverse outcomes among clinical characteristics and serum biomarkers at week 24.

Clinical characteristics	N = 204	Univariate		Multivariate		
		MAOs	*P*	HR	95% CI	*P*
Gender						
Male	148 (72.5%)	56 (37.8%)	0.34	0.95	0.56–1.63	0.84
Female	56 (27.5%)	24 (42.9%)		1.00		
Age at diagnosis						
<17 years	43 (21.1%)	17 (39.5%)	0.87	1.04	0.56–1.81	0.90
≥17 years	161 (78.9%)	63 (39.1%)		1.00		
Disease duration						
<3 years	70 (34.3%)	26 (37.1%)	0.91	1.39	0.79–2.43	0.25
≥3 years	134 (65.7%)	54 (40.3%)		1.00		
Disease type						
Ileitis type	29 (14.2%)	10 (34.5%)	0.75	0.89	0.36–2.20	0.80
Ileocolitis type	146 (71.6%)	59 (40.4%)		1.16	0.61–2.43	0.66
Colitis type	29 (14.2%)	11 (37.9%)		1.00		
Disease behavior						
Penetrating type	46 (22.6%)	24 (52.2%)	0.05	2.39	1.23–4.65	<0.01
Stricturing type	80 (39.2%)	32 (40.0%)		1.41	0.76–2.67	0.28
Inflammatory type	78 (38.2%)	24 (30.8%)		1.00		
Anal lesions						
Present	129 (63.2%)	52 (40.3%)	0.97	1.24	0.74–2.10	0.42
Absent	75 (36.8%)	28 (37.3%)		1.00		
History of smoking						
Present	41 (20.1%)	17 (41.5%)	0.95	0.87	0.47–1.51	0.63
Others	163 (79.9%)	63 (38.7%)		1.00		
Immunosuppressants						
Present	36 (17.6%)	15 (41.7%)	0.82	1.05	0.57–1.82	0.87
Absent	168 (82.4%)	65 (38.7%)		1.00		
CRP at week 24						
≥0.5 mg/dL	46 (22.5%)	26 (56.5%)	<0.01	1.90	1.12–3.16	0.02
<0.5 mg/dL	158 (77.5%)	54 (34.2%)		1.00		
Alb at week 24						
<3.8 g/dL	45 (22.1%)	28 (62.2%)	<0.01	2.20	1.26–3.77	<0.01
≥3.8 g/dL	159 (77.9%)	52 (32.7%)		1.00		

Alb: albumin; CI: confidence interval; CRP: C-reactive protein; HR: hazard ratio; MAOs: major adverse outcomes (hospitalization related to worsening of Crohn’s disease, surgery, and discontinuation due to treatment failure); TNF: tumor necrosis factor.

## Discussion

Following the STRIDE guidelines^5^ and the CALM study^6^, tight control management (treat-to-target strategy) of CD based on biomarkers has gained wide acceptance. Fecal calprotectin is now considered as the most reliable biomarker; however, it is not allowed in routine clinical practice in Asian and some Western countries. The present study suggests that tight control management may help avoid hospitalization, surgery, or discontinuation of anti-TNF therapy in Japanese CD patients with different susceptibility genes and clinical characteristics from Western countries. Even without monitoring fecal calprotectin, we may improve the disease course using only serum biomarkers (CRP and Alb) that can be used in routine clinical practice. Based on upper and lower quartiles, CRP < 0.5 mg/dL and Alb ≥ 3.8 g/dL were optimal standards for tight control management.

Serum CRP has been used as a marker of disease activity in patients with CD^7,8^. However, CRP is a non-specific marker, and often increases in other infectious or inflammatory diseases unrelated to the gastrointestinal tract. In some studies, CRP levels were not associated with the disease activity in small intestinal lesions in patients with CD^7,12^. Despite the lack of strong correlation between CRP and intestinal inflammation, there are no alternative biomarkers for use in routine clinical practice. However, in the present study, CRP level at weeks 8 and 24 predicted the subsequent clinical course including hospitalization and surgery. In Asian settings, use of serum CRP alone may allow for tight control management of CD. In a study on Asian patients with quiescent CD, elevated CRP was found to be associated with poor outcomes^13^.

The optimal standard of CRP for management of disease activity is unclear. In the present study, optimal standard of CRP was set at <0.5 mg/dL based on the upper quartile, rather than at the normal reference level of <0.3 mg/dL for Japanese subjects. Results of multivariate analysis also suggested that CRP < 0.5 mg/dL was an effective biomarker of tight control. During multivariate analysis, inclusion of CRP < 0.3 mg/dL instead of CRP < 0.5 mg/dL did not generate a meaningful model. This may indicate that strict normalization of CRP is not required for tight control of disease activity. In the large multicenter CALM study^6^, CRP > 5 mg/L (CRP > 0.5 mg/dL) was used as one of the criteria for treatment failure in agreement with the present study.

Among serum biomarkers, Alb has also been shown to be associated with treatment course. Patients with low serum Alb at the start of anti-TNF therapy exhibited a tendency to relapse or require surgery earlier than that in patients with high serum albumin^14,15^. This tendency was thought to be due to high clearance of anti-TNF agents in the presence of low serum albumin. The degree of clearance of anti-TNF agents cannot be proven in this study because measurement of serum anti-TNF trough level is not covered under the health insurance system in Japan; however, our results suggest that serum Alb as well as CRP may be an important target for tight control.

In the present study, the optimal standard of serum Alb was set at ≥3.8 g/dL rather than at the normal reference level of ≥4.0 g/dL for Japanese subjects. On multivariate analysis, Alb ≥ 3.8 g/dL was also found to be an effective biomarker of tight control. During multivariate analysis, however, use of Alb ≥ 4.0 g/dL instead of Alb ≥ 3.8 g/dL did not identify any predictive factors. Although the extent of the lesion cannot be quantified, it might be difficult to perfectly normalize serum Alb level in patients with extensive disease or in those with a history of small intestinal resection. To predict the subsequent clinical course, strict normalization of serum Alb may not be required.

In recent years, the efficacy of fecal calprotectin has been reported mainly in Western countries^16,17^. Fecal calprotectin was shown to accurately mirror intestinal inflammation, and to correlate well with prognosis^18,19^. Unfortunately, it is not allowed in Asian and some Western countries. Our present study illustrates that it is possible to manage disease course with readily available blood tests. However, since the sensitivity and specificity of fecal calprotectin have been reported to be higher than CRP or other biomarkers^16,18,20^, a future prospective cohort study should include fecal calprotectin as one of the indices of tight control in Asian settings.

In addition to serum and fecal biomarkers, disease behavior was associated with future disease course in the present study. Penetrating type disease has often been identified as a risk factor for poor prognosis^11^. In general, most medical treatments are not very effective in patients with progressive disease including penetrating type disease. Inflammatory type disease, the so-called window of opportunity, might be the most suitable target for accelerated medical therapy^21,22^, although not actually proved in this study.

We acknowledge several limitations in the present study. First, this was a single-center retrospective study. Second, neither age at diagnosis nor anal lesions which have often been found to be risk factors of poor prognosis^11,23,24^ were identified as risk factors for MAOs. In our gastroenterology division, the majority of patients are adults, while the percentage of younger patients (<17 years) is comparatively low. On the other hand, in Japan as well as in our gastroenterology division, the rate of CD patients with anal lesions is quite high^25,26^. These biased proportions may have influenced our results.

In conclusion, tight control management may help avoid MAOs. In Asian settings where fecal calprotectin is not allowed, CRP < 0.5 mg/dL and Alb ≥ 3.8 g/dL were the best candidate markers of tight control management. If we achieve tight control with optimization of anti-TNF therapy or additional immunomodulators, it might lead to improvement of disease course or better prognosis.
